# In Silico Assessment of the Lipid Fingerprint Signature of ATP2, the Essential P4-ATPase of Malaria Parasites

**DOI:** 10.3390/membranes12070702

**Published:** 2022-07-12

**Authors:** Mario López-Martín, Pedro Renault, Jesus Giraldo, José Luis Vázquez-Ibar, Alex Perálvarez-Marín

**Affiliations:** 1 Biophysics Unit, Department of Biochemistry and Molecular Biology, School of Medicine, Universitat Autònoma de Barcelona, 08193 Bellaterra, Spain; mario.lopez@uab.cat; 2 Institut de Neurociències, Universitat Autònoma de Barcelona, 08193 Bellaterra, Spain; pedro.renault@uab.cat (P.R.); jesus.giraldo@uab.cat (J.G.); 3 Laboratory of Molecular Neuropharmacology and Bioinformatics, Unitat de Bioestadística, Universitat Autònoma de Barcelona, 08193 Bellaterra, Spain; 4 Unitat de Neurociència Traslacional, Parc Taulí Hospital Universitari, Institut d’Investigació i Innovació Parc Taulí (I3PT), Institut de Neurociències, Universitat Autònoma de Barcelona, 08193 Bellaterra, Spain; 5 Instituto de Salud Carlos III, Centro de Investigación Biomédica en Red de Salud Mental, CIBERSAM, 08193 Bellaterra, Spain; 6 Institute for Integrative Biology of the Cell (I2BC), Université Paris-Saclay, CEA, CNRS, 91198 Gif-sur-Yvette, France

**Keywords:** P-type ATPase, *Plasmodium*, flippases, coarse-grain molecular dynamics, lipid–protein fingerprinting, modelling

## Abstract

ATP2, a putative type 4 P-type ATPase, is a phosphatidylinositol-4-phosphate (PI4P)-regulated phospholipid transporter with an interesting potential as an antimalarial drug target due to its conservation across *Plasmodium* species and its essential role in the life cycle of *Plasmodium falciparum*. Despite its importance, the exact mechanism of its action and regulation is still not fully understood. In this study we used coarse-grained molecular dynamics (CG-MD) to elucidate the lipid–protein interactions between a heterogeneous lipid membrane containing phosphatidylinositol and *Plasmodium chabaudi* ATP2 (PcATP2), an ortholog of *P. falciparum* ATP2. Our study reveals structural information of the lipid fingerprint of ATP2, and provides structural information on the potential phosphatidylinositol allosteric binding site. Moreover, we identified a set of evolutionary conserved residues that may play a key role in the binding and stabilization of lipids in the binding pocket.

## 1. Introduction

In addition to the building blocks of membranes, phospholipids are also signaling molecules that control the development and differentiation processes during the intraerythrocytic cycle of *Plasmodium* species, the parasite that causes malaria. For instance, erythrocyte-derived lysophosphatidylcholine controls *P. falciparum*’s intraerythrocytic cycle by repressing the parasite’s sexual differentiation [1]. Moreover, a tight control of phosphatidic acid homeostasis is necessary for the parasite’s egress and subsequent invasion of a new red blood cell [2]. In addition, most of the genes encoding membrane transport proteins involved in lipid transport and in the maintenance of membrane lipid asymmetry are essential for the parasite [3]. One of these essential transporters is ATP2, a putative type 4 P-type ATPase (also called aminophospholipid-transporting P-type ATPase or lipid flippase). In eukaryotes, P4-ATPases maintain the asymmetric distribution of lipids in the membranes, necessary for many important physiological processes, including vesicle budding and protein trafficking [4]. While the pivotal role of ATP2 is still unknown, a recent study has linked the activity of ATP2 with the uptake of the erythrocyte’s hemoglobin into the parasitic digestive vacuole, a fundamental process for parasite progression [5]. ATP2 has also recently emerged as a potential candidate for antimalarial drug targeting since in vitro evolution followed by whole genome analysis has situated the *P. falciparum* ATP2 as the potential target of two antimalarial drugs [6]. Recently, by using the heterologously-produced ATP2 ortholog encoded by the mouse malaria model *P. chabaudi*, PcATP2 (~60% sequence identity to the *P. falciparum* ATP2), it was possible to identify ATP2′s potential Cdc50 β-subunits [7], mandatory proteins for trafficking the majority of lipid flippases to the final membrane destination and, sometimes, for functional activity. Moreover, two phospholipid substrates of PcATP2, phosphatidylserine and phosphatidylethanolamine, were also identified by functional assessment of recombinant PcATP2, as well as the functional upregulatory effect of phosphatidylinositol 4-phosphate [7], a lipid known to be indispensable during the intraerythrocytic development of *P. falciparum*. As ATP2 is highly conserved among *Plasmodium* species, PcATP2 represents a fair functional and structural paradigm of *Plasmodium* ATP2.

In view of the relevance of lipid–protein interactions for flippases’ membrane trafficking, stabilization, and activity, knowledge on the nature of the interacting lipids and the specific residues involved in the interaction is key towards the potential design of efficient antiparasitic strategies. Here, we use coarse-grain molecular dynamic simulations to assess and identify lipid–protein interactions in PcATP2. Using model membranes of asymmetric composition, we aim to fingerprint the lipids and the lipid-binding cavities in PcATP2, which may provide key information for the function and regulation of this transporter, and its therapeutic exploitation. From the perspective of the membrane protein’s structural biology, this approach allows insight to be gained on lipid–protein interactions that can help develop understanding of the structure–function relationships in a potential antimalarial target.

## 2. Materials and Methods

PcATP2 homology modelling. The PcATP2 model is described in a previous study [7]. Briefly, the membrane topology was predicted using the TOPCONS algorithm [8]. The two large intracellular domains (ICD) were independently modeled in MODELLER [9] using 6KG7, 6ROH, 1IWO (ICD1), and 6KG7, 6ROH, 1IWO, 4HQJ, 5MPM (ICD2) as models. Transmembrane regions were modeled using 6K7G and 6ROH as templates in USCF Chimera [10] by alpha carbon molecular replacement. PcATP2 sequence was realigned to the ICD1, ICD2, and TMs models using MAFFT [11] and manually refined. The resulting alignment was used to build the final PcATP2 model in MODELLER [9] using the individual modelled regions as structural templates.

Coarse-Grained Martini simulations. The PcATP2 model was converted to the Martini 2.2 coarse-grained representation; in addition, an elastic network (ElNeDyn22) was used to preserve the protein stability and its secondary structures [12,13]. The protein model was inserted into a membrane using the CHARMM-GUI Martini Maker web server [14,15]. The inner leaflet composition consisted of phosphatidylcholine (POPC), phosphatidylethanolamine (POPE), phosphatidylserine (POPS), sphingomyelin (POSM), and phosphatidylinositol (POPI), while the outer leaflet contained the same lipids, except for POPI. Both leaflets contained approximately 300 lipids each (Table 1). The systems were solvated with Martini water and the charge was neutralized with NaCl to a final concentration of 150 mM. A control system was also built with the same membrane composition but excluding PcATP2. In both cases, four replicates of each system with identical characteristics were run individually.

Each replicate was energy minimized and equilibrated using the default CHARMM-GUI protocol for Martini 2.2 [16]. Briefly, a two-step minimization of 5000 steps each was carried out using the steepest descent algorithm. The first step was performed with soft-core potentials, and for the second step standard hard-core potentials were used. A 5-step equilibration was performed using an NPT ensemble with an increasing integration step (1, 5, 10, 15, and 20 fs, respectively) and decreasing positional restraints in the lipid heads and backbone beads of the protein for a total equilibration time of 4.75 ns. This 5-step equilibration protocol has been extensively tested for membrane and protein–membrane systems [15,17] and allows gradual relaxation of the solvent and the lipids around the protein, as well as relaxation of the lipid tails, prior to the production run by slowly decreasing the restraints in each step. During this, equilibration temperature was kept at 310 K using a v-rescale thermostat, while the Berendsen barostat was used to maintain the pressure at 1 bar. The reaction field and potential shift Verlet methods were used to calculate electrostatics and Van der Waals interactions, respectively. To ensure the system was in equilibrium during the production run, we plotted the temperature, density, and potential energy of the system for all four replicates to monitor the stability of the system (Figure S1).

A 25 µs production for each replica was generated for both the PcATP2–membrane and membrane only replicates using the same parameters as in the equilibration steps but using the Parrinello–Rahman barostat instead. No restraints were applied during the production run. One frame of every 50,000 steps (1 ns time step) of the trajectory was saved for the analysis.

Analysis. Trajectories were PcATP2-centered using the GROMACS built-in *gmx trjconv* tool [18]. The trajectories of the four replicates of each system were concatenated using *gmx trjcat* for the analysis when it was required [18]. Lipid density maps were generated using *gmx densmap* [18]. The relative depletion/enrichment (DE) of lipids around PcATP2 was calculated using *in-house* Python scripts with the MDAnalysis Python package [19,20] applying a contact cut-off distance of 0.55 nm between any bead of PcATP2 and any bead of each lipid species using a similar approach to a previously reported method [21]. The choice of a cut-off distance is dependent on the minimum distance between two beads, which in the case of the Martini 2.2 force field is ~0.50 nm for standard beads (excluding the small beads used for ring structures) [22]. Therefore, the definition of a contact between two beads must be made considering a distance higher but close to 0.5 nm. In our case, the choice of a 0.55 nm cut-off follows this criterion and has also been widely used for the contact analysis between Martini CG beads, including many lipid–protein interaction studies [23,24,25,26,27]. In order to calculate the DE indices, the fraction of each lipid species around 0.55 nm PcATP was calculated for each frame of the trajectory. The ratio between this fraction and the fraction of the corresponding lipid species in the leaflet composition was calculated to obtain the DE index for each species in each frame

$$DE_{X}=\frac{L_{X}\left(0.55\right)/L_{T}\left(0.55\right)}{L_{X}\left(membrane\right)/L_{T}\left(membrane\right)}$$

where *DE* is the depletion/enrichment index, *X* is the lipid species analyzed, *T* refers to all lipid species, and *L* is the number of lipids. Between brackets the area under consideration, 0.55 nm around PcATP2 or the whole membrane. For each species, *DE* mean and standard deviation of the whole trajectory were calculated for each individual replicate. The analysis was performed separately for the outer and inner leaflet of the membrane, which were assigned using the MDAnalysis LeafletFinder algorithm [19,20] on the first frame of the trajectory.

As POPI was shown to accumulate around PcATP2 and phosphatidylinositol is a known regulator of PcATP2 activity [7], it was decided to further explore POPI–PcATP2 interactions in our simulations by analyzing occupancies, residence times, and putative binding pockets.

POPI heads occupancy (*O*), defined as the fraction of frames in which a contact is present, were calculated with MDAnalysis [19,20] in-house Python scripts applying a cut-off distance of 0.55 nm between any POPI head atoms (NC1, NC2, or NC3) and PcATP2

$$O_{A}=\frac{N_{A}\;\left(\mathrm{POPI}\right)}{N_{T}}$$

If *A* is the residue under analysis, *N_A_* (POPI) the number of frames the residue *A* is 0.55 nm or less from any POPI head bead, and *N_T_* is the total number of frames.

POPI residue residence times and binding sites analysis were performed with PyLipID [28] using a dual cut-off of 0.5 and 0.65 nm, analyzing 2500 frames (10 ns time step) of each PcATP2 trajectory. Residence times measure the dynamics of bound lipids and provide an insight on the decay periods of bound lipids to residues/binding pockets, revealing specific (high residence times/long decay periods) or transient (low residence times/short decay periods) interactions. Briefly, PyLipID calculates a dissociation constant (*k_off_)* from a survival time correlation function σ(t)

$$\sigma \left(t\right)=\frac{1}{N_{j}}\frac{1}{T-t}\overset{N_{j}}{\underset{j=1}{\sum }}\overset{T-t}{\underset{v=0}{\sum }}{ñ}_{j}\left(v,v+t\right)$$

If *T* is the length of the trajectory, *N_j_* is the number of lipid contacts between any bead of PcATP2 and POPI, and $\overset{T-t}{\underset{v=0}{\sum }}{ñ}_{j}\left(v,v+t\right)$ is a binary function that adopts a value of 1 or 0 depending on whether the lipid *j* contact lasts from time *v* to time *v + t* or not, respectively. The σ(t) values are calculated for every time step of the trajectory, from *t* = 0 to *t* = *T*, and divided by σ(0) to normalize the survival time correlation function so σ(t)=1 at *t* = 0.

The normalized function is fitted to a biexponential model

$$\sigma \left(t\right)\;∼Ae^{-k_{1}t}+Be^{-k_{2}t}\;\;\;\;\;\;\;\left(k_{1}\leq k_{2}\right)$$

where the first term ($Ae^{-k_{1}t}$) models long decays of POPI relaxation, and the second term ($Be^{-k_{2}t})$ models the short decays. *A*, *B*, *k*_1_, and *k*_2_ are constants. From this model, *k*_1_ is taken as the dissociation constant *k_off_*. PyLipID calculates the residence time (*τ*) as

τ=1koff

To calculate binding sites for a given lipid species, PyLipID detects clusters of residues interacting at the same time with a common lipid molecule. Briefly, for each PcATP2 residue a distance vector is calculated containing the distances to each POPI molecule as a function of time. A Pearson correlation matrix is calculated for those residues binding the same lipid molecule. Using this matrix, a weighted network is constructed in which the protein residues are the nodes and the correlation coefficients of pairs of residues are the weights. The Louvain algorithm [29] is then used to decompose the network into communities (i.e., subsets of nodes/residues more densely connected between them than with the rest of the network). Essentially, Louvain finds the best partitions for the network by iterative optimization of the modularity (*Q*), which is a measure of the quality of the network partitions by comparing the density of the edges within a community and the rest of the network

$$Q=\frac{1}{2m}\underset{i,j}{\sum }\left[A_{ij}-\frac{k_{i}k_{j}}{2m}\right]\partial \left(c_{i},c_{j}\right)$$

where *m* is the sum of all edges in the network, *A_ij_* is the edge weight between nodes *i* and *j*, *k_i_* and *k_j_* are the sum of the weights of the edges attached to the nodes *i* and *j*, respectively; *c_i_* and *c_j_* are communities of nodes *i* and *j*, respectively, and ∂ is a Kronecker delta function that $\partial \left(c_{i},c_{j}\right)=\{\begin{array}{c} 0\;if\;i\neq j\\ 1\;if\;i=j \end{array}\;$.

Each optimization iteration is separated in two phases. After assigning each node to its own community, modularity is optimized by placing each node to the community that increases the modularity the most. This is repeated until no modularity increase is possible. In the second phase, all nodes within the same community are collapsed into a new node and a new network is created. This network is then used to start a new iteration. Iterations are repeated until no changes in the modularity can be observed. The result of this community analysis is a set of nodes (binding sites). Occupancies and residence times are then calculated for the binding sites, allowing identification of those with more stable interactions with POPI.

Residue evolutionary conservation of PcATP2 was performed with ConSurf [30,31] using the default settings. ConSurf conservation scores, PcATP2 residue occupancy by POPI heads and POPI residence time, and POPI-predicted binding pockets were mapped to the all-atom PcATP2 structure b-factor column.

Data were plotted using in-house Python scripts and Seaborn [32,33]. Molecular graphics of PcATP2 were generated using UCSF ChimeraX [34].

## 3. Results and Discussion

In this study, we computationally analyzed the lipid–protein interactions of PcATP2, a close *Plasmodium* ortholog of the essential *P. falciparum* P4-ATPase, PfATP2. Live fluorescent imaging of the *P. berghei* ATP2 ortholog localizes ATP2 at the parasite’s plasma membrane (PPM) [35], situated a few nanometers away from the parasitophorous membrane at the interface parasite/host during the intraerythrocytic stage. In our simulations, we embedded PcATP2 in an asymmetric membrane composed of five different lipid species using coarse-grained molecular dynamic simulations, where the outer leaflet mimics the luminal leaflet of the PPM and the inner leaflet of the cytoplasmic-faced leaflet. All these lipids have been previously identified in a lipidomic analysis of isolated *P. falciparum* parasites from infected erythrocytes [36,37]. PC and PE are the majority species accounting for nearly 50% of the total lipids of the parasite’s membranes. Sphingomyelin (SM) is the third most abundant lipid, representing ~15% of the total membrane’s lipids. This lipid is important for building and maintaining the tubulovesicular network of membranes, a system used by the parasite to traffic its own proteins and then remodel the parasitized red blood cell membrane [38]. Finally, phosphatidylserine (PS) and phosphatidylinositol (PI) represent, respectively, ~6% and ~5% of all phospholipids present in the parasite’s membranes [36,37]. Interestingly, the membrane of the apicoplast, an internal organelle present in apicomplexan parasites such as *Plasmodium* is enriched in PI, reaching up to ~15% of total phospholipid content [37].

**Figure 1:**
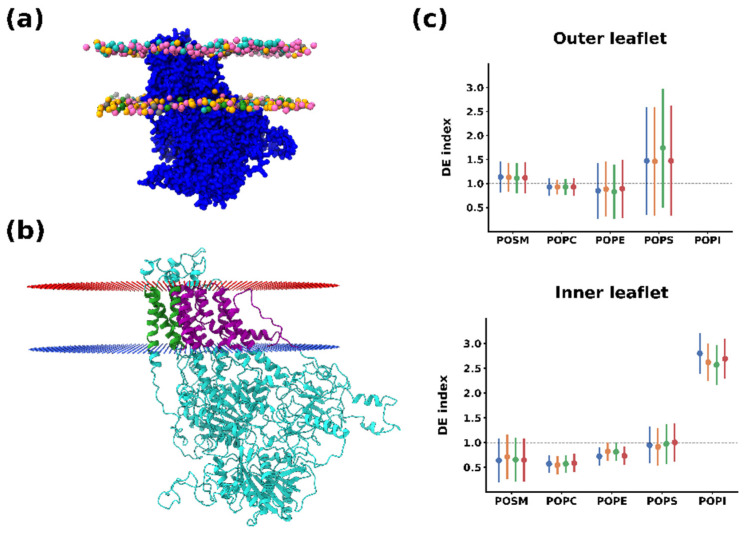
(**a**) System set-up. Lateral view. Each lipid species is colored distinctively (POPC: pink, POPE: orange, POPS: grey, POSM: cyan, and POPI: green). Only lipid phosphate groups are shown. (**b**) All-atom cartoon representation of PcATP2. Protein orientation in the lipid bilayer determined by the PPM server [39] is indicated by red (corresponding to the outer/luminal leaflet) and blue dots (inner/cytoplasmic leaflet). Transmembrane regions of helices 7, 8, and 10 are colored in green. The remaining transmembrane regions are colored in purple. (**c**) Average depletion/enrichment (DE) of lipids as the ratio between the proportion of each lipid species around 0.55 nm of PcATP2 and the ratio of the same species in the membrane composition. Each individual replicate is represented for each lipid with a different color. Error bars represent standard deviations across the whole simulation time. The black dashed line (DE = 1) indicates a homogeneous distribution of the depletion/enrichment index. Values under 1 indicate lipid depletion around PcATP2, while values over 1 indicate an enrichment.

Figure 1a and Table 1 show the system set-up for the simulations. In this asymmetric membrane system, the outer leaflet contained mostly phosphatidylcholine (POPC) and sphingomyelin (POSM), with minor amounts of phosphatidylethanolamine (POPE) and phosphatidylserine (POPS). The inner leaflet, on the other hand, contained higher amounts of POPE and POPS, as well as phosphatidylinositol (POPI), which generates a negative charge at the cytoplasmic side and is also a species that has been involved in the modulation of PcATP2 activity [7]. In the middle of the membrane we embedded the coarse-grained PcATP2 model, which was obtained from a previous study [7]. After 25 μs of MD simulation, we analyzed the whole molecular dynamic trajectory to characterize the lipid shell formed around the protein and establish the unique lipid fingerprint of PcATP2.

**Figure 2:**
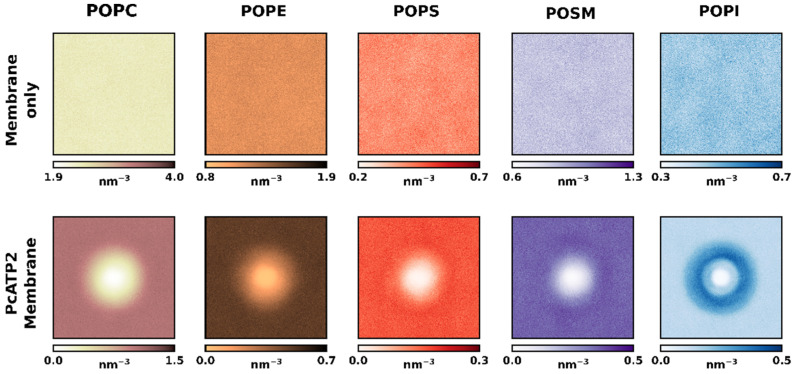
Density maps (in lipids nm^−3^) of each lipid species of both leaflets across the membrane plane of the simulation box for both the control (without PcATP2) membranes (**top**) and the PcATP2 systems (**bottom**).

Figure 1b shows the average relative lipid depletion/enrichment around PcATP2 with respect to the membrane bulk across the whole simulation duration using a 0.55 nm distance cut-off between the lipid and protein beads. In the outer leaflet, we observed a net enrichment of POPS, the only negatively charged lipid present in the initial composition of this leaflet. Interestingly, after the simulation we observed a higher variability in the lipid composition of the inner leaflet surrounding PcATP2. This leaflet is consistently enriched with POPI while the presence of the other lipid species are depleted, with the exception of POPS, which remains approximately at the same proportion as in the membrane bulk. On the other hand, in the inner leaflet POPC displayed the highest degree of depletion around PcATP2. In terms of lipid enrichment through the simulation time, the enhancement of POPS’s presence around the protein was highly variable (Figure S2), while POPI’s enrichment at the inner layer of PcATP2 reached its maximum very early in the simulation and remained stable during the whole simulation for all four replicates (Figure S3). The density plots in Figure 2 that illustrate the redistribution of the different lipid species around PcATP2 compared to empty membranes clearly demonstrate that this redistribution (enrichment or depletion) is driven by PcATP2–lipid interactions, rather than spontaneous lipid–lipid interactions. Similarly, radial distribution functions of the lipids around PcATP2 also reveal a similar enrichment pattern (Figure S4).

The enrichment in the anionic lipids (especially PIs) around the integral membrane proteins has been extensively reported before by molecular dynamic simulations in a wide variety of membrane proteins, including GPCRs, ion channels, and membrane receptors, among others [21,40,41,42]. Indeed, CG-MD simulations using highly complex membrane compositions with more than 60 different species have shown that, despite revealing unique lipid fingerprints for each protein, there is a general trend across most proteins to shell themselves in PIs, along with other negatively charged lipids, generally PS or PA [21]. In our computational approach, we reached similar conclusions using a simple membrane system containing only five different lipid species. This suggests that this simplistic membrane system is able to provide a proper lipid environment to the PcATP2 in order to generate accurate predictions, thus reducing computational requirements without compromising the biophysical properties of the system.

The lipid head group is the main determinant of substrate specificity in P4-ATPases. Phosphatidylserine, one of the most prevalent substrates of P4-ATPases across different species [43], is also one of the putative physiological substrates of PcATP2 [7]. Phosphatidylinositol, on the other hand, has been extensively characterized in simulation and experimental studies across different membrane proteins [21,44,45]. PIs are involved in a wide variety of processes in the cell membrane including protein trafficking and anchoring. Moreover, they are signaling molecules with the capacity to modulate the activity of integral and membrane-associated proteins. The inositol head group can either be non-phosphorylated or mono-, bi-, or tri-phosphorylated (PIPs) at positions 3, 4, or 5 of the ring by different kinases, leading to seven different PIPs with unique subcellular membrane distribution [46]. Interestingly, phosphatidylinositol 4-phosphate (PI4P) is able to upregulate the ATPase activity of PcATP2 in the presence of PS or PE lipid substrates [7], an observation previously reported in other PcATP2 homologs, such as the yeast flippase Drs2p [47,48]. PI4P is an essential lipid for the malaria parasite found at the plasma membrane and at the Golgi apparatus in all stages of the erythrocytic cycle [37]. The inhibition of PI4P synthesis completely blocks the development and progression of the parasite inside the erythrocyte [49].

**Figure 3:**
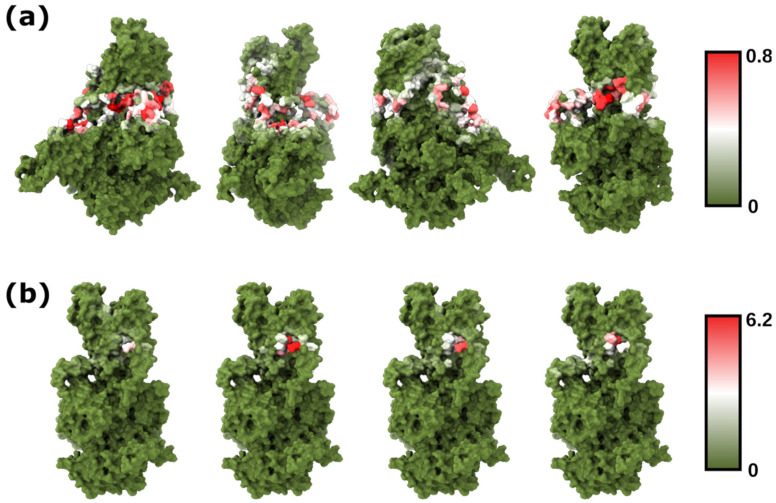
(**a**) Total occupancy of the PcATP2 residues by POPI head groups, represented as percent of time the residue is in contact (≤0.55 nm) with the inositol group, for the combined time of all four replicates. (**b**) Residence times (in μs) of PcATP2 residues with POPI, for each of the four replicates. The selected plane for visualization contains the residues with the highest residence times, located in the putative PI4P/POPI binding site.

The cryo-EM structure of Drs2p in its active form revealed a putative binding site for PI4P between the TMs 7, 8, and 10 [43,50]. Interestingly, most of the Drs2p residues that establish contact with PI4P are well-conserved in PcATP2 [7]. To further evaluate the robustness of our method and gain more structural insights about the PI binding sites of PcATP2, we used PyLipID to analyze the protein–lipid interactions and characterize putative POPI binding sites [28]. By using this more in-depth analysis, we observed that, despite POPI molecules being well-distributed across the transmembrane residues of PcATP2 exposed to the inner leaflet of the lipid membrane (Figure 3a), the residence times of bound POPI lipids are relatively low (<1 μs) in most of the positions (Dataset S2). Indeed, further analysis of each replicate comparing PyLipID occupancies and the residence times of each residue revealed that, despite observing a weak positive correlation between both variables, most residues with high (>0.5) occupancies displayed relatively low (<1 μs) residence times (Figure S6). Residues with the highest (>2 μs) residence times, on the other hand, tended to also possess high occupancies, but there was also variability across residues and replicates. Interestingly, the highest residence times in all four simulations of replicates were located in in the predicted PI4P binding pocket of PcATP2 (Figure 3b and Dataset S2) [7]. Specifically, some of the highest residence times of POPI within the predicted PI4P binding pocket, and consistent in the different replicates, were for residues Trp1288, His1295, Lys1340, Ala1404, and Arg1407, conserved in Drs2p as well as in all *Plasmodium* ATP2 orthologs, along with residues located around those, such as Tyr1208, Tyr1279, Phe1280, Asn1292, Phe1294, Ser1359, and Asp1408 (Dataset S2).

**Figure 4:**
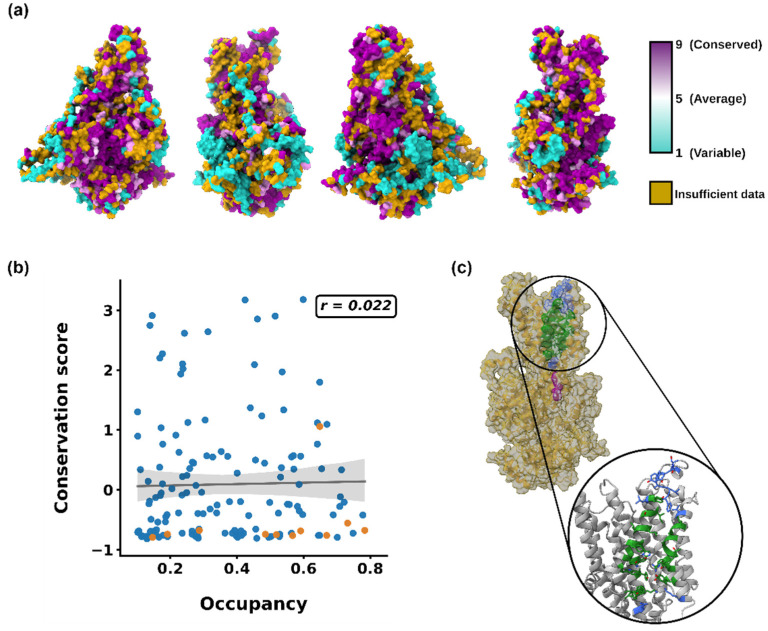
(**a**) ConSurf conservation scores for PcATP2 mapped onto the protein structure. Golden residues represent residues with insufficient data to assign a conservation score. (**b**) Correlation plot between occupancies and conservation scores. Orange dots represent residues of the predicted POPI binding site. Regression line with confidence intervals is represented in black. The Pearson coefficient value (*r*) is shown in the top right. (**c**) Predicted POPI binding site in all-atom representation. Residues common to all four replicates’ (core BP) predictions are colored in green. Residues predicted in only some of the replicates (extended BP) are represented in blue. In purple are Tyr1423 and His1424, the conserved key residues that confer binding specificity for PI4P in Drs2p.

Following these observations, we decided to further analyze the POPI–PcATP2 stable interaction sites by calculating putative binding pockets using PyLipID. The four replicates showed similar binding sites, with the binding pocket with the highest residence time being consistent across replicates and also located between TM7–8–10 (Figure 4c), consistent with our previous analysis and experimental data [43]. To further investigate the conservation of key residues of the POPI binding pocket, we performed a ConSurf conservation analysis of PcATP2. The results revealed some hot spots of conserved residues across PcATP2, including many of the POPI binding site (Figure 4a). Indeed, a correlation plot between occupancies (>0.1) and conservation scores revealed that, despite there not being any correlation between conservation and occupancy, the key residues of the pocket were well-conserved, independently of their occupancies (Figure 4b). Consistent with all these analyses, when looking at the relationship between residence times and conservation, most of the high residence time (>1 μs) residues belonged to the POPI binding pocket and were well-conserved. Moreover, the residues of the binding pocket with low residence times (<1 μs) also possessed a good degree of conservation (Figure S5). Altogether, these results suggest that PcATP2 could have a similar PI4P allosteric activation site to that experimentally characterized in Drs2p [43], and this could be well-conserved across other PcATP2/Drs2p orthologs with a similar allosteric modulation mechanism.

Interestingly, structural and experimental data in Drs2p revealed that residues Tyr1235 and His1236 confer the specificity for PI4P binding by interacting with the inositol-4-phosphate group of PI4P, and the subsequent activation of the ATPase activity of the transporter [43]. These two residues are highly conserved in *Plasmodium* orthologues of Drs2p and are present in PcATP2 as Tyr1423 and His1424. Consistently, ConSurf conservation scores showed that both these residues were highly conserved across all analyzed sequences (Dataset S1). However, in our PcATP2 model the secondary structure in the corresponding sequence region is not a helix but is instead an extended, disordered conformation. Indeed, in Drs2p the helix switch domain is proposed to change its conformation upon PI4P binding from an extended conformation in the inactive state of Drs2p to a fully helical structure, which also induces a movement of this region closer to the binding site [43]. The interaction between the negatively charged PI4P phosphate group with Tyr1235 and His1236 stabilizes the binding of the lipid and the helix structure, triggering the release of the autoinhibitory C-terminal domain [43]. Our PcATP2 model likely represents an autoinhibited conformation where Tyr1423 and His1424 are located far from the predicted PI4P binding pocket, and therefore no interactions between the lipid headgroup of POPI and residues Tyr1423 and His1424 are observed in our simulations. Moreover, the elastic network in the coarse-grained model (ElNeDyn) does not allow changes in secondary structures, as they are fixed by defining additional harmonic bonds [51]. Therefore, the set of conformational changes proposed to occur upon PI binding cannot be observed in our current approach. Nevertheless, as with Drs2p, in this autoinhibited conformation we observe the stabilization of the glycerol backbone of POPI through the interactions with positively charged residues, mainly Arg1407. Indeed, density maps of the Cryo-EM structure of the autoinhibited conformation of Drs2p reveals that other lipids can bind to this pocket with less selectivity than PI4P [43].

In our study we only used non-phosphorylated phosphatidylinositol (POPI in Martini 2.2) as a representative of this lipid species. Despite the lack of phosphorylation in the inositol group, POPI shows stable interactions in specific regions of PcATP2, specifically the predicted binding pocket. Nonetheless, a more extensive study using different PIs species mixed together could help an understanding to be gained of which phosphorylation state, if any, is preferred to interact with the putative binding site of PcATP2. Moreover, the use of all-atom MD or a more flexible coarse-grained model that allows changes in secondary structure, such as SIRAH [52], would be particularly useful to determine whether some of the other residues that play a key role in the PI4P-mediated activation of Drs2p, but do not show a relevant role in POPI binding to the PcATP2-predicted PI4P binding pocket (Tyr1411, Lys1412, Lys1415, Lys1416, Tyr1423, and His1424), also play a role in stabilizing the phosphate group of the inositol group in the *Plasmodium* flippases.

In this study we used a computational approach combining homology modeling of *PcATP2* with coarse-grained molecular dynamics using the Martini 2.2 force field to study lipid–protein interactions of this flippase with a complex asymmetric membrane containing five different lipid species. We observed that PcATP2 creates a local environment rich in anionic lipids, specifically PI. Moreover, we found a conserved PI binding site situated between transmembrane helices TM7, TM8, and TM10 corresponding to the experimentally-proposed PI4P binding site of Drs2p, the *Saccharomyces cerevisiae* ortholog of PcATP2. Our results can help in better understanding the mechanism used by PI4P to upregulate the ATPase activity of PcATP2 in the presence of putative substrates. Moreover, our approach also suggests that the use of coarse-grained systems, as well as simplified membranes with a reduced but relevant selection of different lipid species, can give useful insights into protein–lipid interactions that may be functionally significant, as seen, for example, in the study of lipid-modulated GPCR dimerization [53]. Though coarse graining inevitably leads to a loss of detail in comparison to all-atom models, it allows an important gain in sampling, and therefore it represents a good compromise in the investigation of protein–lipid interactions in PcATP2.

## Figures and Tables

**Table 1:** System initial set-up information, simulation conditions, and membrane composition.

	PcATP2–Membrane		Membrane Only	
Number of replicas	4		4	
Simulation time per replica	25 μs		25 μs	
Time step	20 fs		20 fs	
Number of atoms	32,181		12,591	
Initial box size (*xyz*)	14.68 nm × 14.68 nm × 17.99 nm		14.05 nm × 14.05 nm × 8.57 nm	
	Outer leaflet	Inner leaflet	Outer leaflet	Inner leaflet
POPC	175	100	175	100
POPS	10	36	10	36
POPE	26	103	26	103
POSM	86	21	86	21
POPI	0	45	0	45

## Data Availability

Not applicable.

## Supplementary Materials

The following supporting information can be downloaded at: https://www.mdpi.com/article/10.3390/membranes12070702/s1, Figure S1: Plots of temperature, pressure, density, and potential energy of the systems over the trajectory time. Figure S2: Outer leaflet average depletion–enrichment index; Figure S3: Inner leaflet average depletion–enrichment index; Figure S4: Radial distribution function of each lipid species; Figure S5: Regression plots of the ConSurf conservation score of each PcATP2 residue; Figure S6: Regression plots of the overall contact time of each PcATP2 residue and its residence time with POPI; Dataset S1: PcATP2 ConSurf scores; Dataset S2: PcATP2 POPI PyLipid_Analysis. Figure S7: Structural comparison between PcATP2 model and Drs2p (6ROH). Sequence alignment of the transmembrane regions of PcATP2 and Drs2p [54].
