# Supplementary material for: In Silico Assessment of the Lipid Fingerprint Signature of ATP2, the Essential P4-ATPase of Malaria Parasites
Source: Membranes (Basel). 2022 Jul 12;12(7):702. doi: 10.3390/membranes12070702 (PMC9325222; doi:10.3390/membranes12070702)
Supplement: Supplementary file 1 [file membranes-12-00702-s001.zip › Supplementary_Figures.pdf]

Supplementary Figures

Temperature

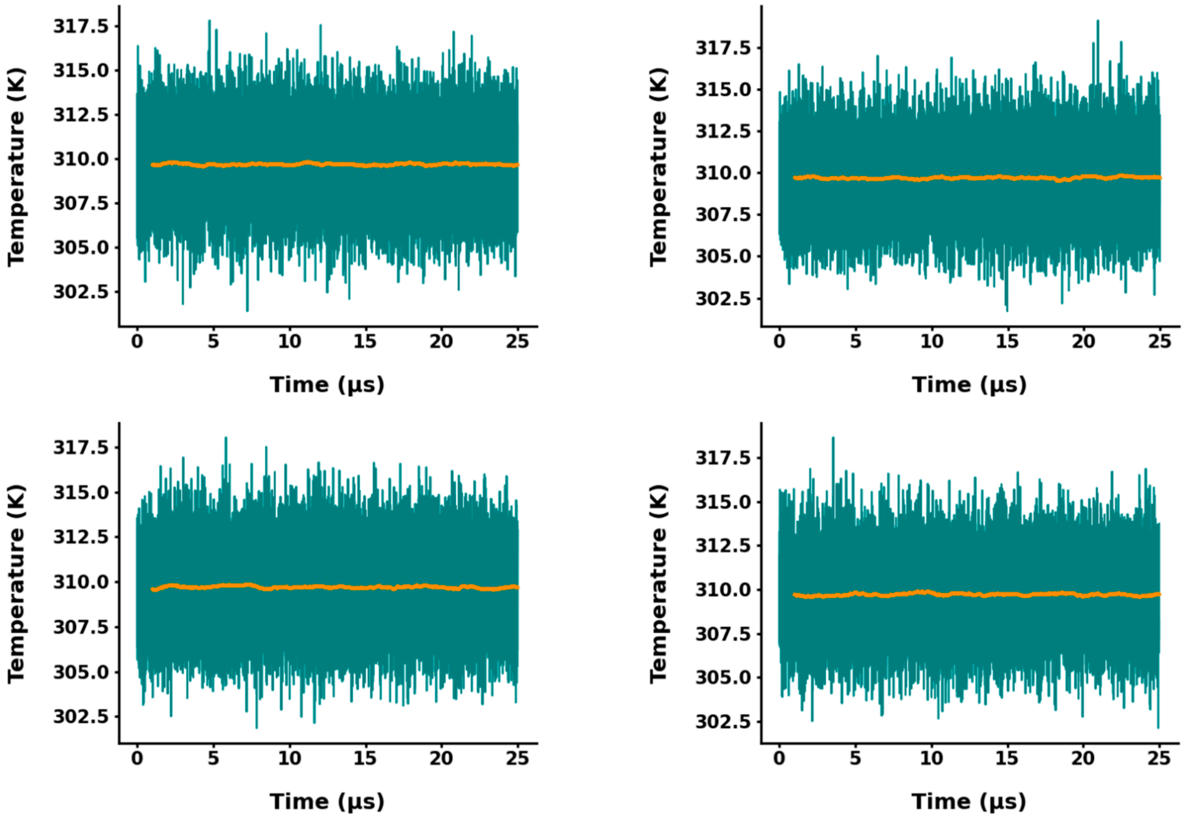

Pressure

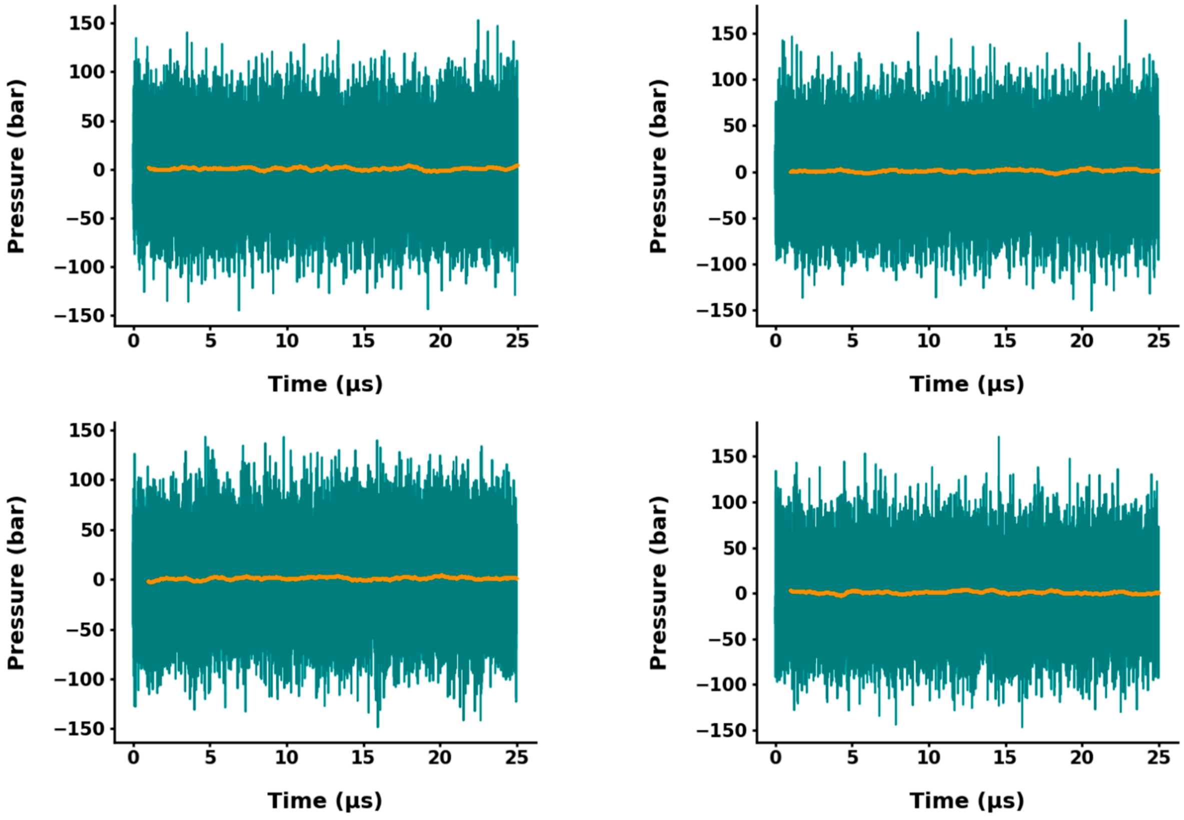

Density

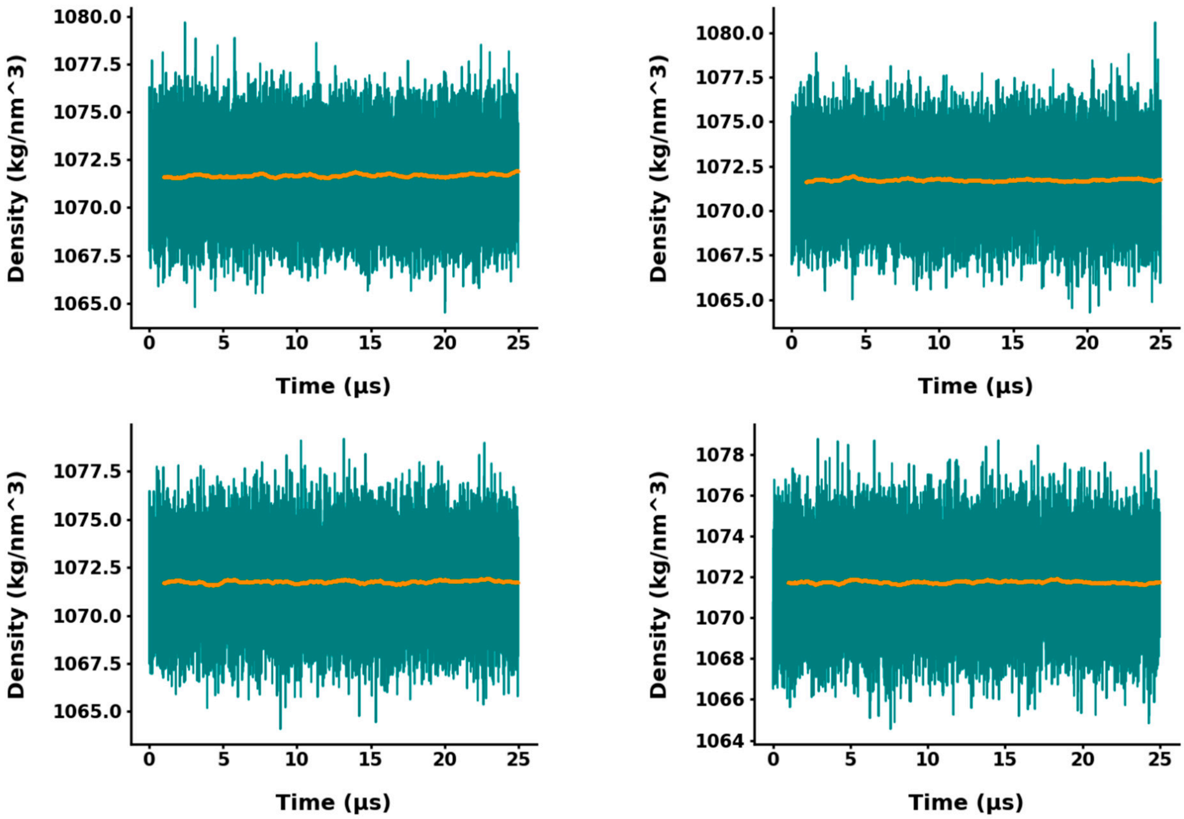

Potential energy

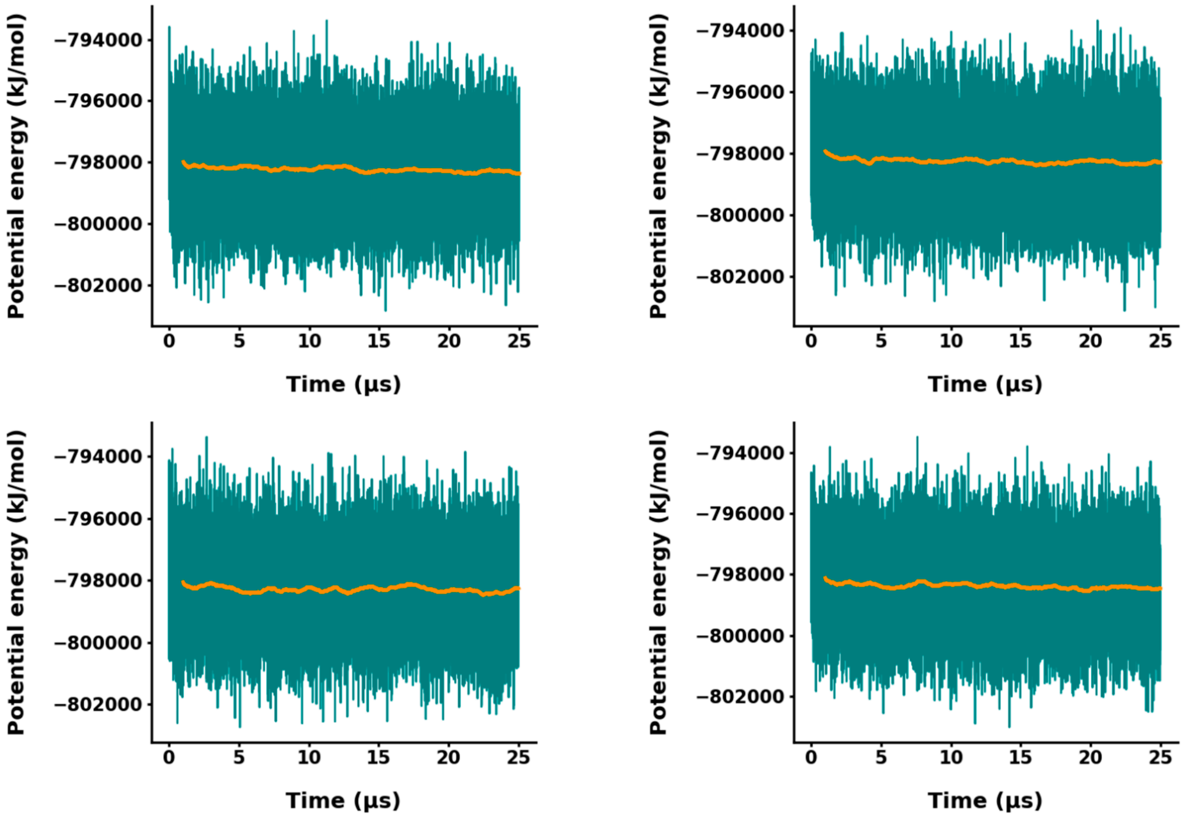

Figure S1: Time evolution of temperature, pressure, density, and potential energy of the systems for the full trajectory of all four replicates. The green line plot represents the values of each variable for every frame (i.e.: 1 data point per nanosecond). The orange line represents the 1000-frame sliding window average.

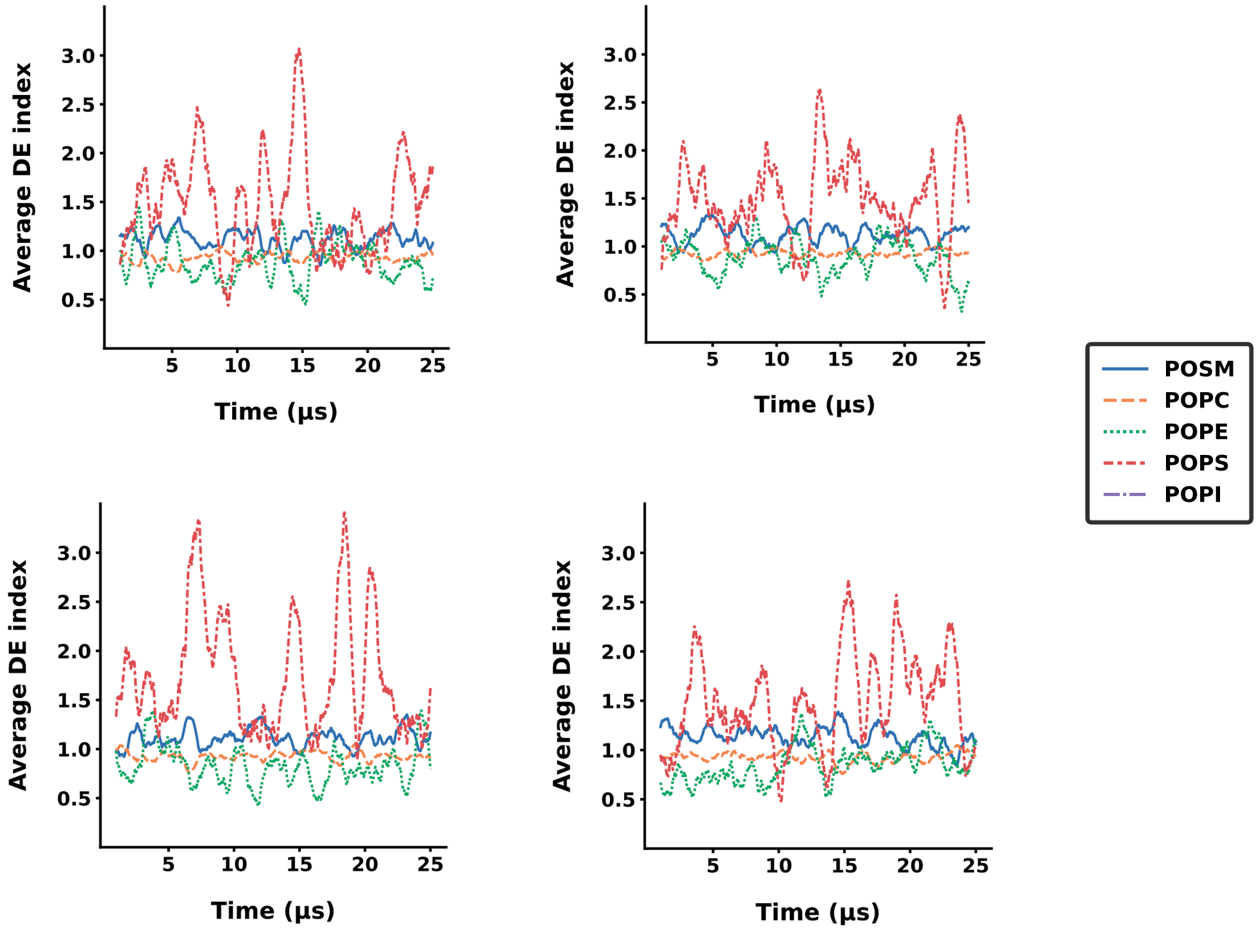

Figure S2: Outer leaflet average depletion-enrichment index (1000 frames sliding window) for each lipid over the system simulation time (25 μs) for each of the four replicates, respectively.

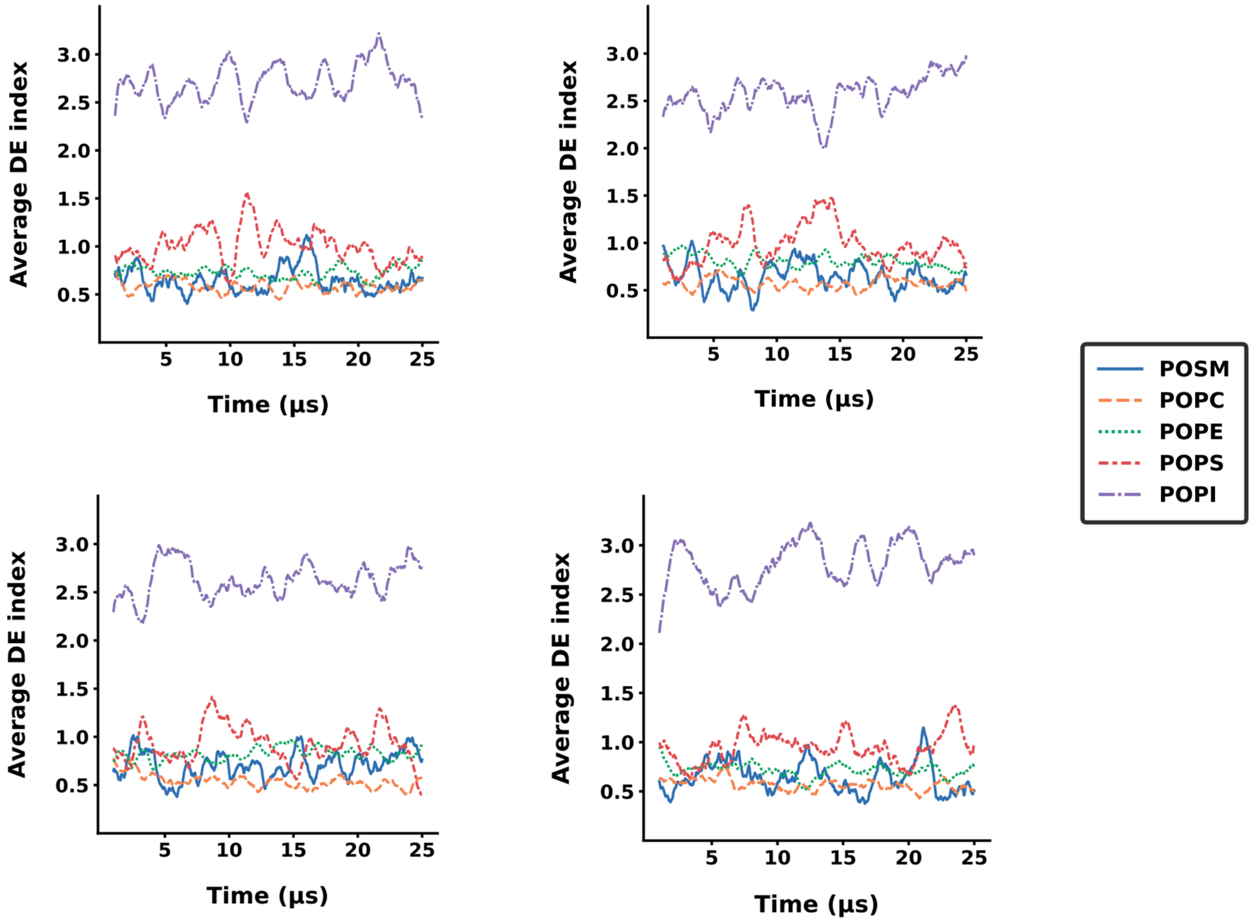

Figure S3: Inner leaflet average depletion-enrichment index (1000 frames sliding window) for each lipid over the system simulation time (25 μs) for each of the four replicates, respectively.

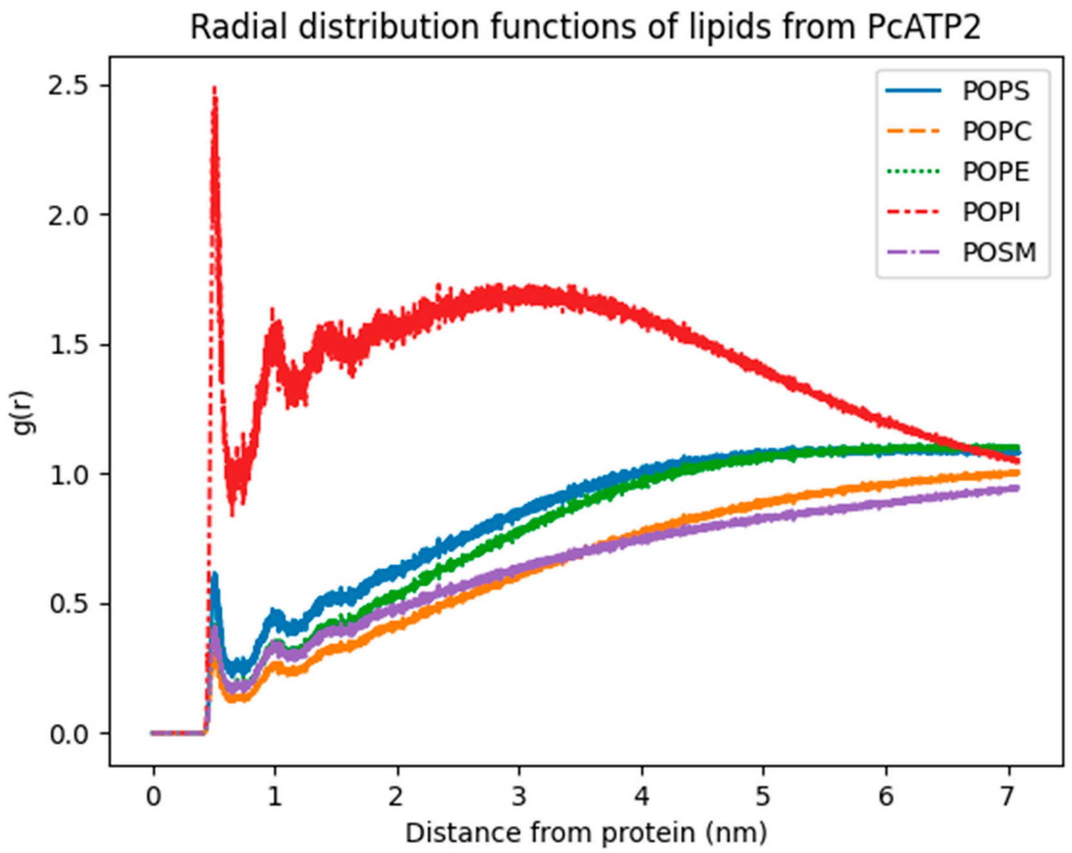

Figure S4: Radial distribution function of each lipid species from PcATP2 for the combined simulation time of all four replicates.

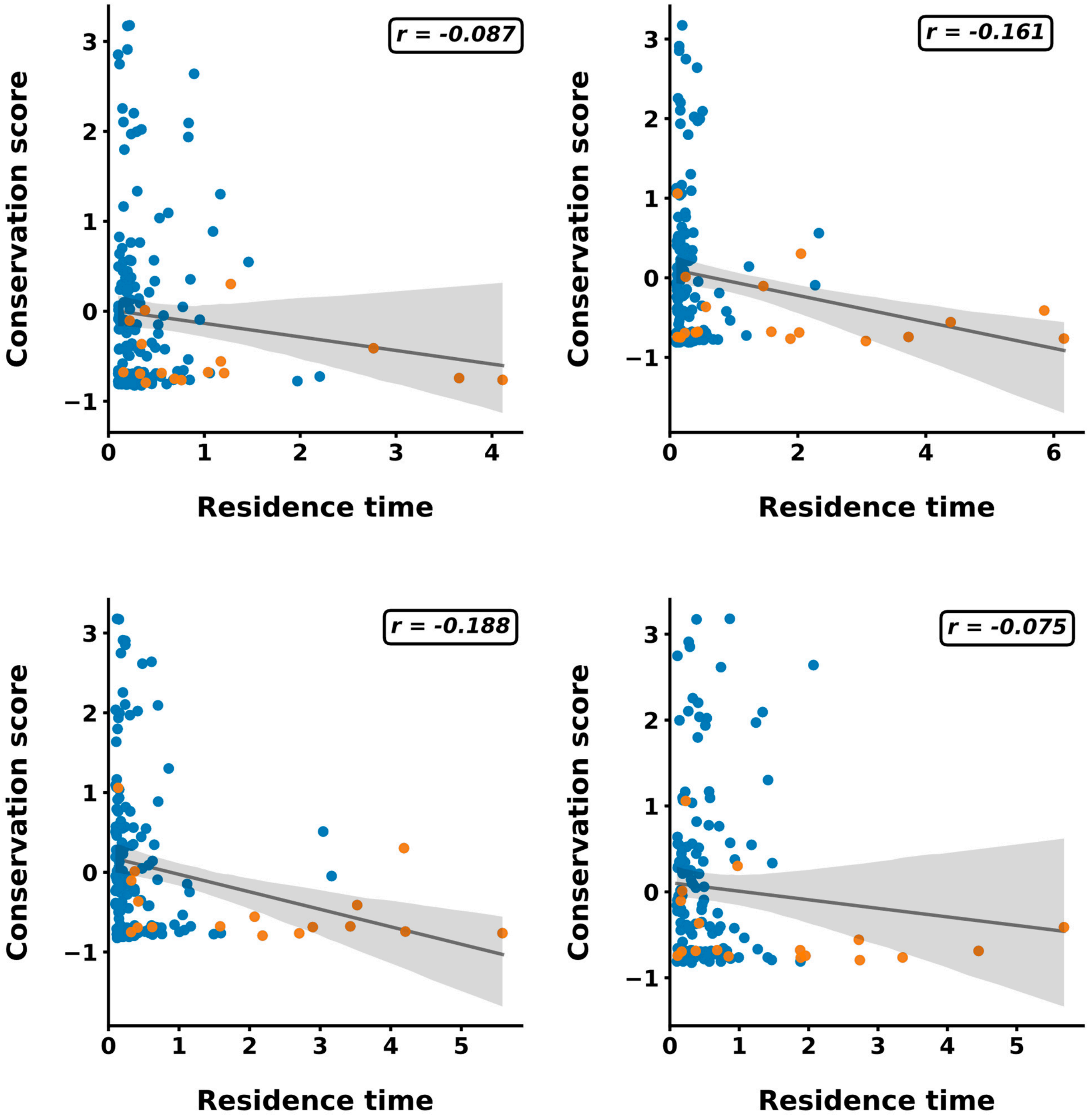

Figure S5: Regression plots for all four replicates, respectively, of the Consurf conservation score of each PcATP2 residue and its residence time with POPI in that replicate. Lower conservation score values represent higher degree of conservation. Orange dots represent residues of the predicted POPI binding site. Regression line with confidence intervals is represented in black. The Pearson coefficient value (*r*) for each replicate is shown in the top right corner of each plot.

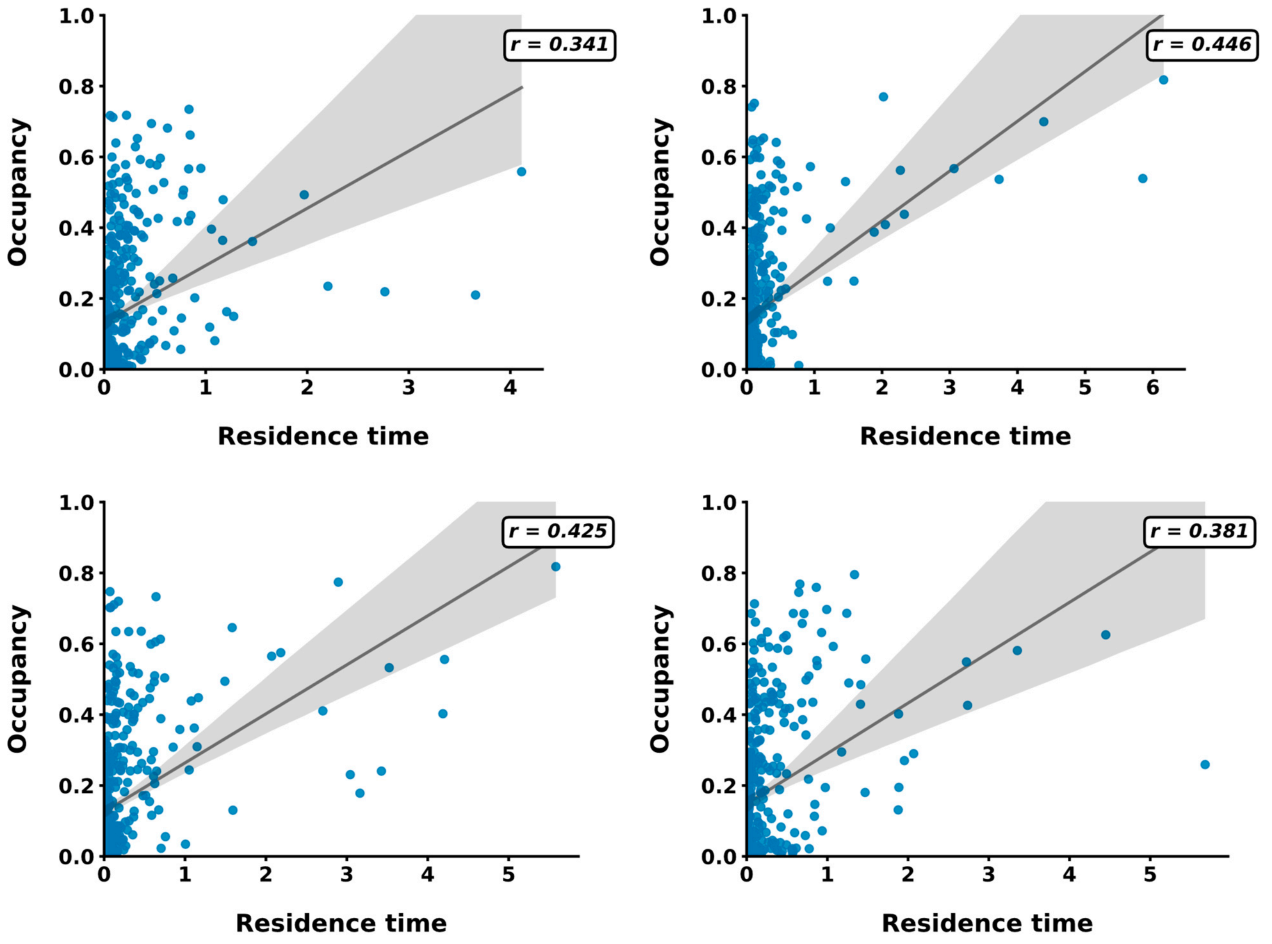

Figure S6: Regression plots for all four replicates, respectively, of the overall contact time of each PcATP2 residue and its residence time with POPI in that replicate. Regression line with confidence intervals is represented in black. The Pearson coefficient value (*r*) for each replicate is shown in the top right corner of each plot.

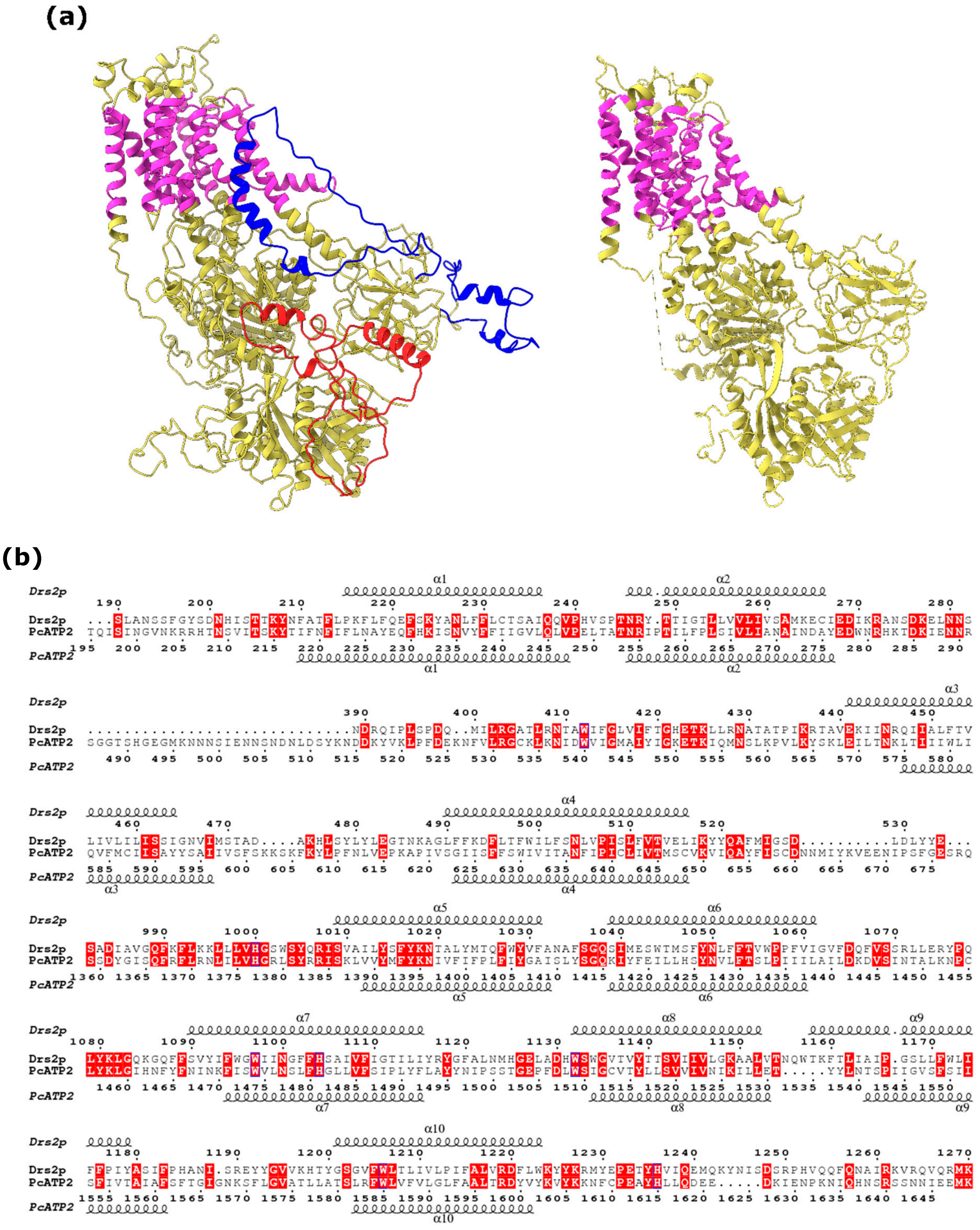

Figure S7: **(a)** Structural comparison between the PcATP2 model (left) and the *Saccharomyces cerevisiae* P4-ATPase Drs2p (PDB id: 6ROH) (right). Transmembrane regions are colored in pink. PcATP2 cytoplasmic domain between TM4 and TM5 missing in Drs2p are colored red. The transmembrane/cytoplasmic domains of PcATP2 between TM2 and TM3 absent in Drs2p are colored blue. **(b)** Sequence alignment of PcATP2 and Drs2p. Regions corresponding to the transmembrane helices common between PcATP2 and Drs2p are depicted (top for Drs2p, bottom for PcATP2). The alignment was performed using ChimeraX [1] *matchmaker* tool using the Needleman-Wunsch sequence alignment algorithm with the BLOSUM-62 matrix and including secondary structure score. ESPript 3 [2] was used to render residue similarity and generate the final figure. Red background indicates conserved residues.

References

[1] E. F. Pettersen *et al.*, “UCSF ChimeraX: Structure visualization for researchers, educators, and developers,” *Protein Science*, vol. 30, no. 1, pp. 70–82, Jan. 2021, doi: 10.1002/PRO.3943.

[2] X. Robert and P. Gouet, “Deciphering key features in protein structures with the new ENDscript server,” *Nucleic Acids Research*, vol. 42, no. W1, pp. W320–W324, Jul. 2014, doi: 10.1093/NAR/GKU316.
